# Structure and dynamics mapping of illicit firearms trafficking using artificial intelligence models

**DOI:** 10.3389/fdata.2025.1648730

**Published:** 2025-09-25

**Authors:** Willy A. Valdivia-Granda

**Affiliations:** Orion Integrated Biosciences Inc., Manhattan, KS, United States

**Keywords:** firearms trafficking, ICCS, BERT, zero-shot prompts, entity resolution, named entities, geocodes, timestamps

## Abstract

Illicit firearms trafficking imposes severe social and economic costs, eroding public safety, distorting markets, and weakening state capacity while affecting vulnerable populations. Despite its profound consequences for global health, trade, and security, the network structure and dynamics of illicit firearms trafficking are one of the most elusive dimensions of transnational organized crime. News reports documenting these events are fragmented across countries, languages, and outlets with different levels of quality and bias. Motivated by the disproportionate impact in Latin America, this study operationalizes the International Classification of Crime for Statistical Purposes (ICCS) to convert multilingual news into structured and auditable indicators through a three-part analytic pipeline using BERT architecture and zero-shot prompts for entity resolution. This analytical approach generated outputs enriched with named entities, geocodes, and timestamps and stored as structured JSON, enabling reproducible analysis. The results of this implementation identified 8,171 firearms trafficking reports published from 2014 through July 2024. The number of firearms-related reports rose sharply over the decade. Incidents increase roughly tenfold, and the geographic footprint expands from about twenty to more than eighty countries, with a one hundred fifty five percent increase from 2022 to 2023. Correlation analysis links firearms trafficking to twelve other ICCS Level 1 categories, including drug trafficking, human trafficking, homicide, terrorism, and environmental crimes. Entity extraction and geocoding show a clear maritime bias; ports are referenced about six times more often than land or air routes. The analysis yielded eighty-five distinct points of entry or exit and forty-one named transnational criminal organizations, though attribution appears in only about forty percent of reports. This is the first automated and multilingual application of ICCS to firearms trafficking using modern language technologies. The outputs enable early warning through signals associated with ICCS categories, cross-border coordination focused on recurrent routes and high-risk ports, and evaluation of interventions. In short, embedding ICCS in a reproducible pipeline transforms fragmented media narratives into comparable evidence for strategic, tactical, and operational environments.

## 1 Introduction

Illicit firearms trafficking is a major driver of violence worldwide, enabling criminal and terrorist groups to weaken state authority, undermine international humanitarian law, and disproportionately impact vulnerable populations. The secretive nature of this trade allows transnational criminal networks to solidify territorial control through violence, confront rival groups, and infiltrate legitimate commercial and logistics networks, leading to consequences that go well beyond national borders. The issue is especially severe in Latin America, a region that, despite making up only 8% of the global population, accounts for roughly 30% of the world's intentional homicides, with about 60% involving firearms. Despite its serious effects on global health, trade, and security, the structure and operational methods of illicit firearms trafficking remain among the most difficult aspects of transnational organized crime to understand.

News reports are a valuable source for understanding the structure and dynamics of firearms trafficking. Articles providing real-time, high-resolution insights into fast-changing developments and crime patterns across diverse geographies (Kazemi et al., 2017; Patil et al., 2022; Wang S. et al., 2023). Despite their potential to improve situational awareness and reveal emerging trafficking trends, combining these reports at a global scale is often vulnerable to misinterpretation due to cognitive biases, translation inaccuracies, and the challenge of contextualizing complex events where multiple crimes frequently occur simultaneously (Muise et al., 2022). To address this challenge, Large Language Models (LLMs) transformer-based deep learning with self-attention mechanisms, have been successfully used to capture relationships between words, phrases, and the context of vast text data (Wang S. et al., 2023; Minaee et al., 2020). Recent advances in domain-adapted LLMs pretrained on extensive multilingual corpora, such as BERT and its variants, DistilBERT, RoBERTa, amBERT, XLM-R, LaBSE, and AraBERT, have demonstrated significant effectiveness in processing information for security-oriented applications and across diverse linguistic environments such as Arabic, Pashto, and Spanish (Yang, 2024; Wang H. et al., 2023).

Models such as DarkBERT (Jin et al., 2023), CyberBERT, BERTimbau (Souza et al., 2020), BERTweet (Nguyen et al., 2020), and CT-BERT (Müller et al., 2023) have been deployed to analyze content from social media and dark web communities, with particular emphasis on firearm-related violence, civil unrest, and emerging health risks. These implementations have facilitated the creation of advanced crime-monitoring frameworks, including AI4Crime, CEASEFIRE, and INSPECT (Cani et al., 2024), which integrate natural language processing techniques such as named entity recognition, geotagging, and relation extraction. Collectively, these systems and other approaches illustrate the potential of transformer-based architectures to uncover latent patterns in illicit activities and to enhance the situational awareness of law enforcement and public policy institutions (Fares and Abd Elaziz, 2025; Zhang and Lu, 2025). However, while tools using LLMs often achieve over 90% accuracy and have significantly enhanced the capacity of analysts and law enforcement agencies, most implementations lack a standardized and interoperable framework for classifying and interpreting crime-related outputs, particularly when integrating data across multiple languages, jurisdictions, and information sources. Existing systems rely on custom taxonomies or informal, keyword-based categories (e.g., “drug trafficking,” “terrorism,” “cybercrime”), which can lead to inconsistencies in threat labeling and category duplication.

The International Classification of Crime for Statistical Purposes (ICCS), developed by the United Nations Office on Drugs and Crime (UNODC), provides a standardized framework for classifying criminal offenses across countries and over time. Structured as a four-level taxonomy that covers 11 broad crime categories at Level 1 to more than 230 detailed categories at Level 4, the ICCS enables both broad aggregation and fine-grained analysis of crime data (Bisogno et al., 2015). While originally designed to harmonize official statistics, its potential, and the objective of this work is to explore its broader potential as a tool for structuring unstructured, multilingual crime-related information drawn from the news. Media reports often rely on heterogeneous and ambiguous terminology, complicating systematic analysis. By embedding ICCS codes into automated analysis pipelines, large language models (LLMs) can map journalistic accounts of crime onto a globally recognized taxonomy, reducing ambiguity, enhancing comparability, and enabling coherent cross-national monitoring of emerging threats. Applied to phenomena such as illicit firearms trafficking, this approach offers a structured lens for tracing actors, networks, and geographic patterns in near real time, bridging the gap between fragmented media narratives and standardized international crime statistics.

## 2 Methods

### 2.1 Data acquisition, preprocessing, and classification

**Figure 1:**
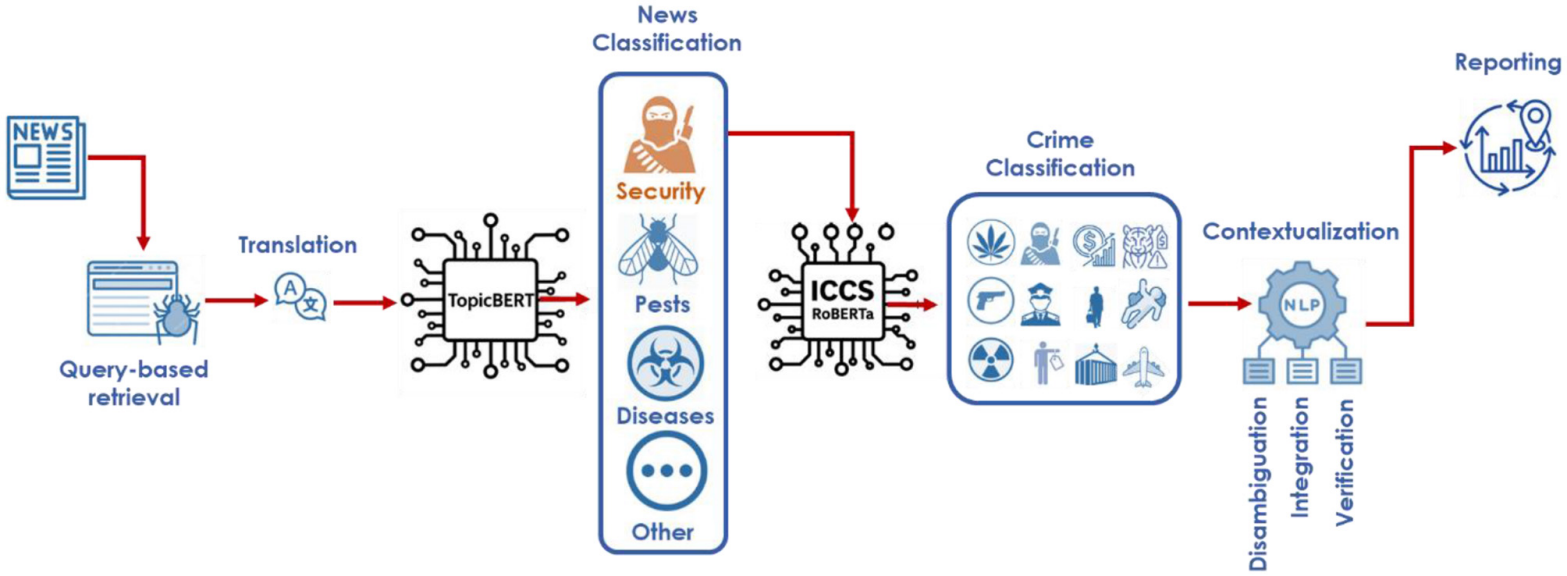
Workflow of the multilingual crime analysis pipeline. News articles are retrieved and translated before being classified by TopicBERT into thematic domains (Security, Pests, Diseases, Other). Security-related texts are further categorized by ICCS–RoBERTa using the UN International Classification of Crime for Statistical Purposes (ICCS). Outputs are contextualized through NLP modules for disambiguation, integration, and verification, and then aggregated into structured reports for trend analysis and monitoring.

We built a Python-based ingestion pipeline that issues targeted queries to public and subscription application programming interfaces and to RSS feeds to gather news articles. The system collected relevant reporting published from 2014 through 2024 under the platform's terms of service. For processing, we captured the article's full text together with source metadata, language tags when available, and timestamps; the narrative prose was not persisted after processing. We cleaned and normalized text (Unicode normalization and lowercasing) and removed exact and near duplicate articles. We used the model's native tokenizer and did not remove stop words or other tokens before modeling; SpaCy tokenization was used only for named entity recognition. Articles in languages other than English were machine-translated to English with Google Translate to standardize downstream classification (Johnson et al., 2016). For the transformer classifier, we used a three-step classification approach (Figure 1):

**a) Topic Classification:** As the first analytic step, we applied topic modeling to the preprocessed corpus to surface three dominant themes. Using BERTopic with sentence embeddings from all MPNet Base V2, a unigram vocabulary (n-gram range 1,1), and min_topic_size = 10, the model produced coarse clusters that we interpreted as pests, diseases, and security. We used these topic assignments only for triage: articles in the security theme were routed to the next categorical classification stage. At the same time, non-security items were excluded from downstream crime analysis.

**b) ICCS Classification:** Crime categorization using the ICCS hierarchical architecture used a single-label RoBERTa base uncased focused exclusively on security-related new events, performing hierarchical categorization in accordance with Levels 1 and 2 of the International Classification of Crime for Statistical Purposes (ICCS), as established by the United Nations Office on Drugs and Crime (UNODC) (Bisogno et al., 2015; Sanh et al., 2019). These categories included 0901 Acts involving weapons, explosives, and other destructive materials, and their subcategories 090121 trafficking of firearms and ammunition, 090122 trafficking of other weapons or explosives, and 090129 other weapon-related trafficking. We instantiated RobertaForSequenceClassification with hidden and attention dropout of 0.10 and tokenized with byte-level BPE, applying truncation and padding to a maximum of 256 tokens. Optimization used AdamW with a learning rate of 2.0 × 10^−5^, weight decay 0.01, a linear schedule with 10 percent warmup, gradient clipping at 1.0, mixed precision, and batch sizes of 16 for training and 32 for evaluation. Training ran for up to five epochs with early stopping after one epoch without improvement on development macro F1. Class imbalance was handled with class-weighted cross-entropy with weights from inverse class frequency; when a minority class fell below 25 percent in the training split, we enabled minority up-sampling in the data loader. Model selection used development macro F1, and any decision thresholding was tuned on the development portion and then applied unchanged to the held-out split in each fold.

### 2.2 Geospatial resolution and entity linking

During processing, we retained only factual elements in structured JSON: named entities (organizations, locations, commodities, ports, airports), event timestamps, security labels with model scores, geocodes, and source identifiers. This data minimization kept less than five percent of each article by character count, enabling efficient spatiotemporal retrieval and aligning with fair use and platform terms. Entities, especially geographic references and criminal organizations, were extracted with SpaCy multilingual NER and a fine-tuned Flair tagger to improve accuracy across languages and regions; geographic mentions were geocoded with GeoNames. Organizational resolution used zero-shot classification with GPT 3, prompted by hypotheses such as “[ENTITY] is a known transnational criminal organization,” with disambiguation aided by country context and frequent co-occurrences. We built a monthly incident time series from publication dates and internal timestamps for each ICCS category co-reported with firearms trafficking. Pairwise Pearson correlations were computed between categories, and converted these to a distance matrix as 1 – r and performed hierarchical clustering in Genesis with the options Average linkage WPGMA, cluster genes (rows, ICCS categories), and cluster experiments (columns, ICCS categories) (Sturn et al., 2002). The heat map displays correlation values on a −1 to 1 scale (green to red) with row and column dendrograms determining order.

## 3 Results

### 3.1 Model performance

The ICCS provides a hierarchical taxonomy, starting with 11 broad crime categories at Level 1, followed by 47 subcategories at Level 2, approximately 162 categories at Level 3, and culminating in 230 specific categories at Level 4. In our evaluation, the classification model achieved a precision of 0.89, a recall of 0.85, and an F1-score of 0.87 at Level 1, and a micro-F1 score of 0.79 across 47 Level 2 categories.

### 3.2 Illicit firearms trafficking structure and dynamics

**Figure 2:**
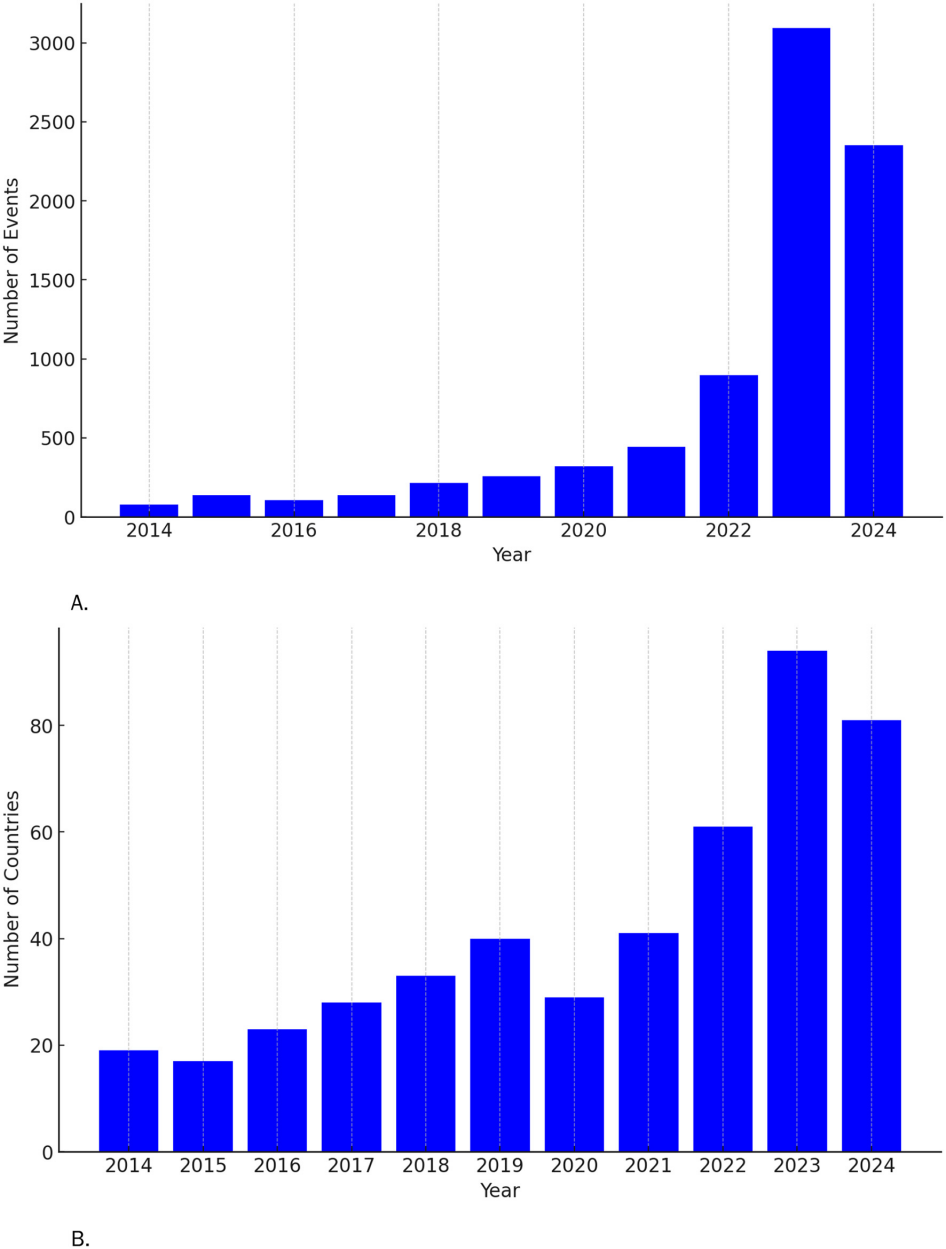
**(A)** Cumulative illicit firearms trafficking reports (2014–2024). **(B)** Geographic coverage over time.

Between March 2014 and July 2024, our analysis classified 8,171 news reports of ilicit firearms trafficking (Figure 2A). In 2014, 20 countries were mentioned in the reports; by 2024, this number had risen to more than 80 (Figure 2B). News reports from Latin America (4,575) frequently referenced other locations operationally linked to the region, including North America (2,458), Europe (1,678), Asia (456), Africa (82), and Oceania (22). Between 2022 and 2023, the average number of news reports on illicit firearms trafficking increased by 155% (Table 1). The leading countries in both South and North America reporting illicit firearms trafficking incidents were Colombia (2,040), the United States (892), Mexico (808), Brazil (544), Ecuador (329), Paraguay (302), Chile (299), Peru (195), Argentina (136), Canada (118), Venezuela (87), Haiti (81), Bolivia (71), and the Dominican Republic (63). In Europe, Spain reported the highest number of incidents (496), followed by France (228), Italy (122), Ireland (121), the United Kingdom (120), and Belgium (82). Firearms trafficking reports mentioned 71 ports of entry or exit in 21 countries. Movements skew toward exits (39) over entries (32) and by mode are 33 maritime, 21 air, and 17 land. Exits concentrate in the United States (17) and Spain (7), while entries cluster in Colombia (6), Peru (6), and Brazil (5), with a maritime bias at gateways like Buenaventura, Santa Marta, Callao, and Porto Velho (Table 2). The NER analysis identified that firearms and ammunition seizures were linked to 41 known transnational polycriminal organizations operating across multiple countries (Table 3). However, these organizations accounted for only 40% of the reported cases, with the remaining reports lacking specific details on the responsible entities.

**Table 1:** Annual variation of firearms trafficking incidents per country.

**Country**	**Count 2022**	**Count 2023**	**Percentage change**
Colombia	590	1,372	132.54
Brazil	66	431	553.03
Ecuador	129	255	97.67
Paraguay	21	212	909.52
Peru	81	201	148.15
Chile	212	192	−9.43
Argentina	63	59	−6.35
Bolivia	7	24	242.86
United States	291	486	67.01
Mexico	243	372	53.09
Canada	32	80	150
Haiti	36	26	−27.78
Dominican Republic	12	25	108.33
Panama	1	11	1,000
Puerto Rico	1	8	700
Guatemala	23	7	−69.57
Honduras	11	7	−36.36
El Salvador	6	6	0
Bahamas	1	5	400
Jamaica	8	5	−37.5
Barbados	2	3	50
Trinidad and Tobago	9	3	−66.67
Russian Federation	7	12	71.43
Spain	172	268	55.81
France	54	97	79.63
United Kingdom	54	97	79.63
Ireland	54	71	31.48
Germany	2	53	2,550
Italy	9	46	411.11
Belgium	19	37	94.74
Slovenia	3	27	800
Serbia	1	24	2,300
Ukraine	22	17	−22.73
Croatia	3	14	366.67
Czechia	1	14	1,300
Netherlands	21	14	−33.33
Portugal	2	12	500
Romania	2	12	500
Poland	2	9	350
Albania	2	8	300
Nigeria	2	25	1,150
Uganda	7	9	28.57
Sudan	2	8	300
South Africa	2	4	100

**Table 2:** POEs with incidents of firearms trafficking.

**Country**	**Port**	**Pathway**	**Route**
Argentina	Port of Buenos Aires	Maritime	Entry
Argentina	Port of Madero	Maritime	Entry
Belgium	Port of Antwerp	Maritime	Exit
Bolivia	El Alto Int.	Aerial	Entry
Bolivia	Viru Viru	Aerial	Entry
Bolivia	Desaguadero Border Crossing	Terrestrial	Exit
Brazil	Rio de Janeiro–Galeão Int.	Aerial	Entry
Brazil	Porto Seguro	Maritime	Entry
Brazil	Port of Porto Velho	Maritime	Entry
Brazil	Foz do Iguaçu Border Crossing	Terrestrial	Entry
Brazil	Tabatinga Border Crossing	Terrestrial	Exit
Brazil	Oiapoque Border Crossing	Terrestrial	Entry
Chile	Valparaiso Marine Port	Maritime	Entry
Chile	Arica Border Crossing	Terrestrial	Entry
Colombia	Alfonso Bonilla	Aerial	Entry
Colombia	El Dorado	Aerial	Entry
Colombia	Barranquilla Port	Maritime	Entry
Colombia	Port of Buenaventura	Maritime	Entry
Colombia	Port of Santa Marta	Maritime	Entry
Colombia	Leticia Border Crossing	Terrestrial	Entry
Ecuador	Guayaquil Airport	Aerial	Entry
Ecuador	Port of Guayaquil	Maritime	Entry
Ecuador	Port of Manta	Maritime	Entry
Haiti	Port of Cap-Haitien	Maritime	Entry
Italy	Port of Reggio Calabria	Maritime	Exit
Mexico	Port of Veracruz	Maritime	Entry
Mexico	Port of Manzanillo	Maritime	Entry
Montenegro	Port of Bar	Maritime	Exit
Netherlands	Port of Rotterdam	Maritime	Exit
Paraguay	Silvio Pettirossi	Aerial	Exit
Paraguay	Guaraní de Minga Guazú	Aerial	Exit
Paraguay	Puente Int. de la Amistad	Terrestrial	Exit
Panama	Port of Panama	Maritime	Exit
Peru	Jorge Chavez	Aerial	Entry
Peru	Jari River	Maritime	Entry
Peru	Paita Marine Terminal	Maritime	Entry
Peru	Port of Callao	Maritime	Entry
Peru	Aguas Verdes Border Crossing	Terrestrial	Exit
Peru	Santa Rosa	Terrestrial	Entry
Peru	Desaguadero Border Crossing	Terrestrial	Entry
Portugal	Port of Lisbon	Maritime	Exit
Portugal	Port of Porto	Maritime	Exit
Spain	Barajas Airport	Aerial	Exit
Spain	Costa del Sol Airport	Airport	Exit
Spain	El Prat Airport	Airport	Exit
Spain	Palma de Mallorca Airport	Airport	Exit
Spain	Port of Denia	Maritime	Exit
Spain	Port of Málaga	Maritime	Exit
Spain	Port of Motril	Maritime	Exit
Trinidad and Tobago	Piarco International	Aerial	Entry
Trinidad and Tobago	Port of Spain	Maritime	Entry
United Kingdom	Birmingham Airport	Airport	Exit
United Kingdom	Gatwick Airport	Airport	Exit
United States	Dallas/Fort Worth	Aerial	Exit
United States	Fort Lauderdale	Aerial	Exit
United States	La Guardia	Aerial	Exit
United States	J.F. Kennedy	Aerial	Exit
United States	Miami Airport	Aerial	Exit
United States	Port Everglades	Maritime	Exit
United States	Port of Bridgeport	Maritime	Exit
United States	Port of Gulfport	Maritime	Exit
United States	Port of Miami	Maritime	Exit
United States	Port of Portland	Maritime	Exit
United States	Del Rio Border Crossing	Terrestrial	Exit
United States	Eagle Pass Border Crossing	Terrestrial	Exit
United States	El Paso Border Crossing	Terrestrial	Exit
United States	Hidalgo/Pharr/Reynosa	Terrestrial	Exit
United States	Laredo Border Crossing	Terrestrial	Exit
United States	Nogales Border Crossing	Terrestrial	Exit
United States	San Ysidro	Terrestrial	Exit
Uruguay	Port of Montevideo	Maritime	Entry

**Table 3:** TCO and main trafficking activities, in addition to firearms trafficking.

**Criminal Org**	**Country**	**Firearms trafficking**	**Human trafficking**	**Cocaine trafficking**	**Marihuana trafficking**	**Illegal mining**	**Wildlife trafficking**	**Timber trafficking**
La Cordillera, Los Compa	Colombia	Yes	No	Yes	Yes	Unknown	Unknown	Unknown
Autodefensas Conquistadores de la Sierra, Los Pachenca	Colombia	Yes	Unknown	Yes	Yes	Yes	No	Yes
La Constru	Colombia, Ecuador	Yes	Unknown	Yes	Unknown	Yes	Unknown	Unknown
Comandos de la Frontera, La Mafia 48, Los Sinaloa, Ex FARC Frente 48	Colombia, Ecuador	Yes	Unknown	Yes	Yes	Yes	No	No
Ejercito de Liberacion Nacional (ELN)	Colombia, Ecuador, Venezuela, Peru	Yes	Yes	Yes	Yes	Yes	Yes	Yes
Coordinadora Arauco Malleco	Chile, Cuba, Argentina,	Yes	No	Yes	Yes	-	No	No
Los Choneros	Ecuador, Peru, Colombia	Yes	Yes	Yes	Yes	Yes	Yes	Yes
Los Lobos	Ecuador, Peru, Colombia	Yes	Yes	Yes	Yes	Yes	Yes	Yes
Los Chone-Killers	Ecuador, Peru, Colombia	Yes	Yes	Yes	Yes	Yes	No	No
Los Tiguerones	Ecuador, Peru, Colombia	Yes	Yes	Yes	Yes	Yes	No	No
Kompania Bello	Albania, Ecuador, the Netherlands, Belgium, Italy	Yes	Yes	Yes	Yes	No	No	No
Los Guardianes de la Trocha, Ronda Campesina El Pueblo	Peru, Brazil	Yes	Yes	Yes	No	Yes	Unknown	Unknown
Fadil Kacanic	Albania, Ecuador	Yes	No	Yes	No	No	No	No
Clan Farruku	Albania, Ecuador, Italy, Belgium, Greece, Portugal, the Netherlands, Spain. Morocco	Yes	No	Yes	No	No	No	No
Youda Marseille mafia	France, Morocco, Spain, Italy, Algeria, Libya	Yes	No	Yes	Yes	No	No	No
DZ Mafia	France, Morocco	Yes	No	No	No	No	No	No
Kraze Baryé	Haiti, Dominican Republic	Yes	Unknown	Yes	Yes	No	No	No
Abastecedores de Ventanilla y Callao	Peru, Ecuador, Turkey	Yes	No	No	No	No	No	No
'Ndrangheta	Italy, Brazil, Panama, Colombia, Peru, Ecuador, Costa Rica, Chile, Bolivia, Paraguay, Uruguay, Germany, Belgium, France, Portugal, Slovenia, Greece, Spain, Romania, Moldova, Luxembourg, United States, United Kingdom, Pakistan	Yes	Yes	Yes	Yes	Yes	No	No
Tren de Aragua	Venezuela, Colombia, Ecuador, Perú, Chile, Brazil, Costa Rica, Mexico, Trinidad and Tobago, Spain, United States	Yes	Yes	Yes	Yes	Yes	No	Yes
Primeiro Comando da Capital	Brazil, Colombia, Peru, Bolivia, Ecuador, Panama, Chile, Paraguay, Portugal, France, Spain, Netherlands, Belgium, Guinea-Bissau, Benin, Nigeria, Cape Verde, South Africa	Yes	Yes	Yes	Yes	Yes	No	Yes
Clan del Golfo	Colombia, Ecuador, Honduras, Panamá, Republica Dominicana, Venezuela, Brazil, Costa Rica, Chile, United States, Spain, Germany, France, Portugal, Poland, Greece, Ireland, United Kingdom, Italy, Albania, Ukraine	Yes	Yes	Yes	Yes	Yes	Yes	Yes
Ex-FARC Front Carolina Ramirez	Colombia, Ecuador, Brazil, Peru	Yes	Yes	Yes	Yes	Yes	Yes	Yes
Ex-FARC Segunda Marquetalia	Colombia, Venezuela, Panama, Ecuador	Yes	Yes	Yes	Yes	Yes	Yes	Yes
Ex-FARC Estado Mayor Central (EMC)	Colombia, Venezuela, Ecuador	Yes	Yes	Yes	Yes	Yes	Yes	Yes
Cartel de Jalisco Nueva Generación	Mexico, United States, Colombia, Ecuador, Dominican Republic, Peru, Brazil, Venezuela, Chile, Bolivia, Paraguay, Argentina, Uruguay, Guyana, Netherlands, Belgium, Italy, Spain, Portugal, Germany, France, the United Kingdom, Ireland, Serbia, Albania, Romania, Slovakia as well as Morocco and Turkey	Yes	Yes	Yes	Yes	Yes	Yes	Yes
Sinaloa Cartel	Mexico, United States, Colombia, Ecuador, Dominican Republic, Peru, Brazil, Venezuela, Chile, Bolivia, Paraguay, Guyana, Argentina, Uruguay, France, United Kingdom, Italy, Spain, Balkans, Lebanon, Turkey, South Africa, Tanzania, Kenya	Yes	Yes	Yes	Yes	Yes	Yes	Yes
Comando Vermelho, Movimiento Sanguinario	Brazil, Colombia, Peru, Ecuador, Bolivia, Paraguay	Yes	Yes	Yes	Yes	Yes	Yes	Yes
Bala na Cara	Brazil, Paraguay, Argentina, Uruguay	Yes		Yes				
Los Trinitariarios	Dominican Republic, Chile, Spain, United States, United Kingdom	Yes	No	Yes	Yes	No	No	No
Los Dominican Don't Play	Dominican Republic, Spain, United States	Yes	Yes	Yes	Yes	No	No	No
400 Mawozo gan	Haiti, Dominican Republic, United States	Yes	Yes	Yes	Yes	No	No	No
Hezbollah	Lebanon, Venezuela, Colombia, Peru, Brazil, Argentina, Paraguay, Iran	Yes	Yes	Yes	Yes	Yes	Yes	Yes
Balkan cartel	Serbia, Colombia, Ecuador	Yes	Yes	Unknown	Unknown	Unknown	Unknown	Unknown
Giorgi-Boviciani Clan	Italy, Colombia, Belgium, the Netherlands, Germany, Australia, Pakistan	Yes	Yes	Yes	Unknown	Unknown	Unknown	Unknown
Škaljarski Clan	Serbia, Peru, Ecuador, Belgium, Montenegro, Spain, Greece, Ukraine, Turkey	Yes	Yes	Unknown	Unknown	Unknown	Unknown	Unknown
Zemun mafia group	Paraguay, Balkans	Yes	Yes	Unknown	Unknown	Unknown	Unknown	Unknown
Bang of Fujian	China, Chile	Yes	Yes	Yes	Unknown	Unknown	Yes	Unknown
Albanian Cartel	Albania, Ecuador	Yes	Yes	Unknown	Unknown	Unknown	Unknown	Unknown
Mocro Maffia	Morocco, Turkey, Colombia, Italy, Spain, Belgium, Netherlands	Yes	Yes	Unknown	Unknown	Unknown	Unknown	Unknown
Kinahan cartel	Ireland, Colombia, Panama, Russia, Dubai	Yes	Yes	Yes	No	No	No	No

### 3.3 Illicit firearms trafficking dynamics and correlation with other crimes

**Figure 3:**
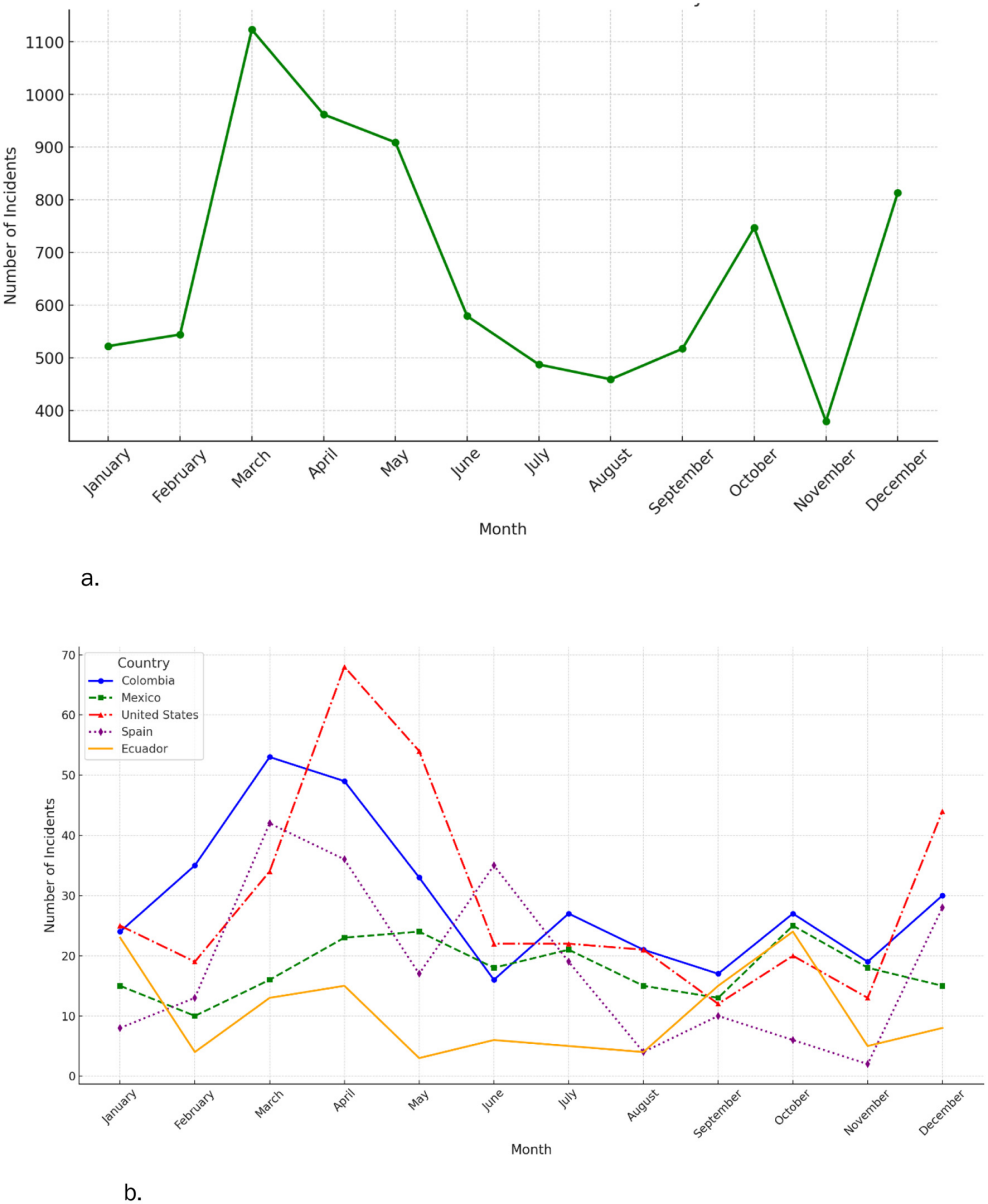
**(a)** Monthly average of events reporting firearms trafficking from 2014–2023. **(b)** Monthly average of incidents reporting firearms trafficking during 2021–2023.

**Figure 4:**
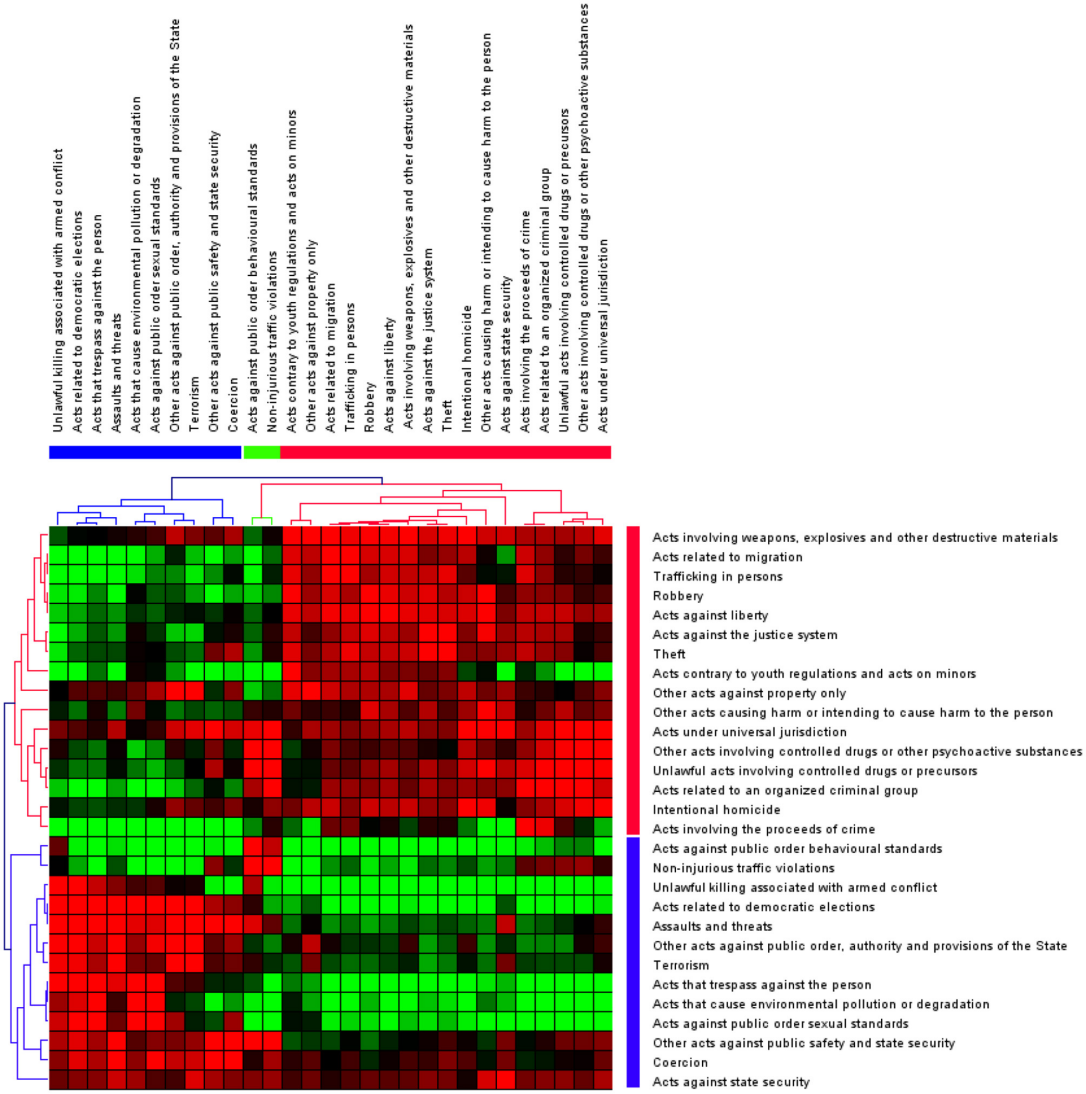
Correlation cluster of ICCS crime categories co-reported with firearms trafficking (2021–2023).

News reports about firearms show seasonal differences, with peaks from March to May (Figure 3a). This pattern persists across countries, as indicated by data from 2021 to 2023 (Figure 3b). Kendall correlations among UN crime categories, extracted from reports from 2021 to 2023, identified strong positive correlations between weapons-related offenses (UN ICCS code 0901) and unlawful acts involving controlled drugs or precursors (UN ICCS code 0661), with a correlation coefficient of 0.75. Similarly, acts under universal jurisdiction (UN ICCS code 1101), including crimes against humanity, show high correlations with intentional homicides (UN ICCS code 0101) (0.74) and drug offenses (UN ICCS code 0661) (0.79), indicating a significant overlap in their occurrence with ilicit firearms trafficking. Intentional homicides also correlate strongly with human trafficking (UN ICCS code 0704) (0.79), highlighting potential interconnections within criminal networks. Conversely, environmental crimes (UN ICCS code 0804) exhibit lower correlations with firearms trafficking and other crimes but a strong correlation with human trafficking. No significant negative correlations were observed, implying that these crimes do not typically inversely relate (Figure 4).

## 4 Discussion

This study presents the first known application of the International Classification of Crime for Statistical Purposes (ICCS) as a foundational taxonomy for training large language models to classify and analyze multilingual news articles on firearms trafficking. By incorporating the ICCS into the LLMs training and analysis workflow, these artificial intelligence systems overcome language barriers, minimize regional disparities in media reporting, and reduce the influence of cognitive biases in news reporting. This enables a structured and interpretable analysis of illicit firearms trafficking across Latin America. The results provided a tempo-spatial quantitative assessment of firearms-related criminal activity, providing detailed insights into the geographic and temporal dimensions of this transnational threat. However, future work requires benchmarking this approach against traditional linear models trained on TF-IDF features and alternative transformer encoders (e.g., BERT-base, XLM-R) under a common training and evaluation protocol to quantify trade-offs in accuracy and efficiency.

**Figure 5:**
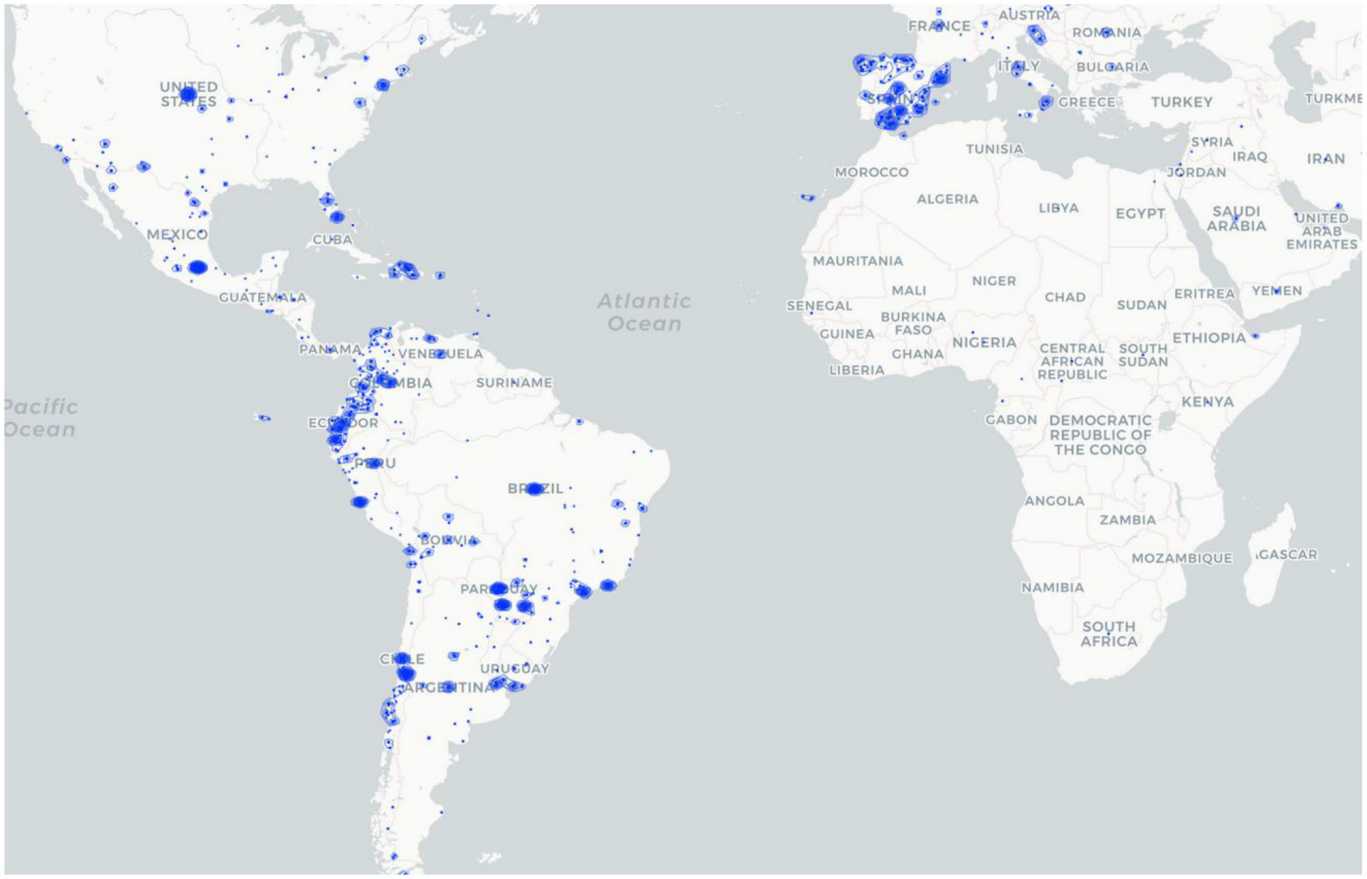
Geolocation of incidents reporting firearms trafficking events.

This analysis of news reports identified a significant surge of illicit firearms trafficking that parallels recent country-specific studies of firearms related to the violence, drug trafficking, and other criminal activities exerted by TCOs' expansion (Vásquez et al., 2023; Dressler and Wolff, 2024; UNODC, 2023; Gandilhon, 2024; Llorente et al., 2024; Le Cour Grandmaison et al., 2024; Álvarez, 2024b; Mires-Agip and Solís-Castillo, 2024; Fabre et al., 2023; Medina, 2020). The cumulative data from 2021 to 2023 patterns and a consistent spike from February to May suggest a seasonal nature to firearms trafficking across several countries. These temporal patterns might be associated with multiple factors, particularly the coca harvesting season, which highlights potential periods for targeted interventions by law enforcement. Although 41 TCOs and 85 POEs were identified in firearms trafficking crimes, 60% of incidents were unattributed to any specific organization, highlighting the complexity and challenges associated with mapping this type of transnational crime. However, the analysis uncovered that although firearms are trafficked via land, sea, and air, maritime ports of entry and exit POEs are cited six times more frequently than airports and land crossings. These quantitative results agree with previous observations about maritime crime (Gandilhon, 2024; Sosnowski et al., 2024). Although further analyses are required to determine primary or secondary POEs, establish the extent of TCOs penetration in transportation networks, and determine the volume of firearms trafficked in hundreds of illegal crossing points through rivers, tunnels, and land crossings. At the same time, we can establish hot spots of firearms trafficking beyond Latin America (Figure 5). Nonetheless, the results of embedding ICCS in a reproducible pipeline transform fragmented media narratives into comparable evidence for strategic, tactical, and operational environments.

Overall, the patterns of firearms trafficking anecdotally reveal three pivotal factors driving the surge in the illicit arms trade in Latin America: the expanding criminal diversification of TCOs, the militarization of TCOs, and the quasi-political transformation of TCOs.

### 4.1 The diversification of TOC

Analyzed news co-reported transnational firearm trafficking with 60 out of 230 other criminal categories defined by the ICCS. Of these, 12 crime categories have strong positive correlations with firearms trafficking, underscoring the deeply intertwined diversity of criminal activities and the need for multidimensional approaches to counter crime that simultaneously impact global health, trade, and security. While human trafficking moderately correlated with firearms trafficking, this crime had a strong correlation with environmental crimes like illicit wildlife, logging, and natural resources trafficking. Firearms trafficking often involves the use of falsified documentation, disguised cargo, and complex transshipment routes that exploit legitimate businesses. Once in circulation, these firearms are utilized to assert control over agricultural supply chains, leading to violent land grabs, displacement of communities, and domination of transport and distribution networks. Specifically, intricate supply routes associated with commodities like fruits, vegetables, timber, salmon, and shrimp are penetrated to covertly transport drugs in exchange for money, drug precursors, and additional firearms (Murillo-Sandoval et al., 2023).

### 4.2 The professionalization of TOCs

Illicit firearms trafficking news reports increasingly highlight the involvement of active and former military and police personnel, as well as ex-combatants from criminal and terrorist organizations. Driven by economic incentives or coercion, these individuals bring specialized expertise in intelligence, counterintelligence, military logistics, combat tactics, and weaponry skills to criminal organizations. Their involvement significantly enhances criminal operations, diversifies their activities, and challenges the integrity and morale of law enforcement and military personnel (Taer and Christenson, 2024; Escobar-Jiménez, 2024; Briceño-León, 2023). This is also one of the main drivers of diverting legally acquired weapons from armed forces and security corporations into the ilicit market.

The militarization of TCOs with more powerful and sophisticated firearms escalates violent disputes of vast territories across various countries and military challenge to armed government forces (Torres, 2024; Gómez-Vega, 2024; Álvarez, 2024a). This is sparking an arms race, not only among criminal groups but also among law enforcement agencies striving to regain regions overrun by organized crime. Due to weak state authority and insufficient law enforcement capabilities, local security and vigilante groups have emerged in Mexico, Colombia, Ecuador, Peru, Haiti, and Brazil. These militias have even forged alliances with criminal organizations to procure firearms, blurring even further the lines between community defense and collaboration with illicit actors.

### 4.3 The quasi-political transformation of TOCs

The firearms trafficking news reports indicate that TCOs continue to gain territorial control and exploit weaknesses of law enforcement, judicial, port, border, and correctional authorities (Briceño-León, 2023). As with terrorist groups, the firepower of TCOs forces governments at the local, regional, and national levels to enter into tacit or formal peace agreements that are profoundly reshaping the security landscape across Colombia, Mexico, Haiti, Ecuador, Brazil, Venezuela, and Bolivia (Llorente et al., 2024; Jański, 2022; Guarin, 2020). During this quasi-political transformation, TCOs accumulate more firearms to challenge the government but face a heightened risk of internal fragmentation and violent territorial disputes influenced by national and international partners or competitors. In some regions, TCOs are increasingly perceived by local civilian populations as de facto authorities, providing essential services and enforcing their justice systems. Criminal governance leads to the perception that official state laws impose only moderate penalties with minimal enforcement. In contrast, the punishments meted out by TCOs are much harsher and almost certain to be enforced. This level of coercion heightened the control of TCOs over local communities and political influence at the regional and national levels.

## 5 Conclusions

ICCS-trained AI models overcome language barriers, regional reporting disparities, and potential cognitive biases to provide a tempo-spatial quantitative analysis of illicit firearms trafficking in Latin America. The results of this analysis show an escalating trend of news reports related to firearms trafficking, with a 155% rise in incidents between 2022 and 2023. While 41 criminal organizations were linked to firearms trafficking, they accounted for only 40% of the cases in new reports. The regional structure and dynamics of illegal firearms trafficking events appear to be driven by the criminal diversification of TCOs into new illicit activities, the militarization of their operations through the acquisition of more sophisticated weaponry, and their pseudo-politicization, which increasingly leverage firepower to influence political negotiations with the government. Correlations between illicit firearms trafficking and diverse crimes, including homicides and human and drug trafficking, point to the convergence of criminal activities with significant implications for global health, trade, and security.

## Data Availability

The raw data supporting the conclusions of this article will be made available by the authors, without undue reservation.
